# The electrochemical detection of bioterrorism agents: a review of the detection, diagnostics, and implementation of sensors in biosafety programs for Class A bioweapons

**DOI:** 10.1038/s41378-021-00242-5

**Published:** 2021-02-10

**Authors:** Connor O’Brien, Kathleen Varty, Anna Ignaszak

**Affiliations:** Department of Chemistry, University of New Brunswick, 30 Dineen Drive, Fredericton, NB E3B 5A3 Canada

**Keywords:** Chemistry, Electronic properties and materials, Biosensors

## Abstract

During the past year, disease has shown us the iron grip it can hold over a population of people. Health systems can be overwhelmed, economies can be brought into recession, and many people can be harmed or killed. When weaponized, diseases can be manipulated to create a detriment to health while becoming an economic burden on any society. It is consequently prudent that easy detection of bioweapons is available to governments for protecting their people. Electrochemical sensing displays many distinct advantages, such as its low limit of detection, low cost to run, rapid generation of results, and in many instances portability. We therefore present a wide array of electrochemical sensing platforms currently being fabricated, a brief summary of Class A bioweapons, and the potential future of bioweapon detection and biosafety.

## Introduction

In the era of COVID-19, it has never been more obvious that disease holds immense power to cause fear and disruption. The intentional spread of an infectious agent is therefore of prominent concern for any nation’s security, health, and economy. In the United States of America, the cost of running biosecurity programs was 1.61 B in the 2019 fiscal year, a year in which no attacks were reported, thus indicating the importance of safety to government and security agencies^1^.

Bioterrorism is defined as the intentional release of biological agents to cause illness or death in people, animals, and plants^2^. Biowarfare has been utilized long before the known existence of microorganisms. The first recorded use of biological warfare occurred in the fourteenth century BC when the Hittites sent diseased rams to weaken their enemies before battle^3^. The advancement of microbiology, the dissemination of information, and the ability to select for specific biological agents marked the beginning of the modern use of biowarfare. Bioweapons were used effectively and efficiently in the colonization of the Americas and in the US civil war. Furthermore, bioweapon programs were effectively established during the Second World War. Between 1990 and 1999, the instances of bioterrorism displayed a fivefold increase when compared with the previous 10 years^4^. In 2001, 2 years after the aforementioned report, the word “bioterrorism” made international headlines due to anthrax attacks at US postal service locations, which resulted in five deaths (Table 1).

**Table 1:** Examples of the use of microorganisms in biowarfare during the past millennia^5^

Date	Examples of the use of microorganisms in biowarfare
Prehistoric times	Melanesian tribesman (actual Vanuatu) used arrowheads contaminated with tetanus
Fourteenth century BC	The Hittite army sends rams infected with tularemia to their enemies
Sixth century BC (Trojan War)	Scythian archers infected their arrows by dipping them into decomposing cadavers and human blood containing *C. perfringens* and C. tetani
1155	Emperor Barbarossa poisons water wells with human bodies in Tortona, Italy
1340 (Hundred Years War)	Jean, Duke of Normandy, cast dead horses over the wall into the besieged the castle of Thun l’eveque, which was captured by the Englishmen
1346	Tartar (Mongol) army catapulted bodies of plague victims over the city walls of Caffa (Feodosia, Ukraine) to attack the Genoese army
1422	Lithuanian army catapulted corpses of those who died in battle, manure and garbage into the town of Karlstein (Bohemia)
1495	Spanish sold wine mixed with the blood of leprosy patients to their French opponents in Naples (Italy)
1500	Pizarro offered variola-contaminated clothing to South American native communities
1650	Polish fire saliva from rapid dogs toward their enemies
1676: Antoine Philips van Leeuwenhoek, commonly referred as “the Father of Microbiology”, identifies microorganisms	
1710	Russian army catapulted bodies of plague victims into Swedish cities in Reval (Estonia)
1763 (French–Indian War)	British offered smallpox-contaminated blankets to Native Americans
1776–1781 (American Revolutionary War)	British attempt to spread smallpox among the continental forces by inoculating civilians fleeing from Boston
1797	The Napoleonic armies flood the plains around Mantua (Italy) to enhance the spread of malaria
1861–1863 (American Civil War)	Confederate troops sold yellow fever and smallpox-infected clothing to Union troops
	Confederate troops contaminate water supplies for the Union forces with animal corpses
End of the 19th century: development of the germ theory of disease and the foundation of microbiology by Louis Pasteur (1822–1895) and Robert Koch (1843–1910)	
1914–1918 (the First World War)	German troops sold horses and mules infected with glanders and anthrax to the Allies
	German troops sold sheep infected with glanders and anthrax to Russia (in Romania)
	German troops sold sheep infected with glanders and anthrax to the Britain and Indian armies
	German troops attempted to spread cholera in Italy and plague in St. Petersburg
1925: The “Protocol for the Prohibition of the Use in War of Asphyxiating, Poisonous or Other Gases and Bacteriological Methods of Warfare”, also referred as the “Geneva Protocol”, was signed (38 signatories and 140 parties)	
1939–1945 (the Second World War)	Japanese army poisoned water wells in Chinese villages to study cholera and typhus outbreaks
	Japanese inoculated prisoners of ware with agents causing gas gangrene, anthrax, meningitis, cholera, dysentery, and plague
1972: The “Convention on the Prohibition of the Development, Production and Stockpiling of Bacteriological (Biological) and Toxin Weapons and on their Destruction”, also referred as the “Biological Weapons Convention” (BWC) was signed (actually has 182 parties)	
2001: The US Patriot Act is signed, providing Federal and national law enforcement officials with enhanced counterterrorism capacities	

In some of the presented cases (e.g., plague during the siege of Caffa, smallpox during the French–Indian War, and yellow fever during the American Civil War) it is difficult to distinguish if the disease spread was due to the intentional release of microorganisms or if it was due to the limited hygienic conditions during the period or the contact between populations with different immunities^5^

Although attacks are infrequent, the WHO has conducted models to display the profound effect a mass-bioterrorism attack could have on a localized population. The simulation predicted that upon the dissemination of anthrax spores in a densely populated area of 1 million individuals, 95,000 deaths and an additional 30,000 hospitalizations would occur. In addition to the death and injuries that would be present, resources to cope with such an attack would be sparse, especially in rural communities^6^. This type of event would require mass changes to medical, societal, and residential infrastructure, thus furthering the case that detection and prevention is preferable to a postattack response.

The ability to detect the agent of infection is a key factor in the success of isolating and managing a biohazard incident^7^. Electrochemical sensing is an ideal platform as testing is generally rapid, easy to use, sensitive, portable, and cost-effective^8^. By measuring the change in current, potential, conductance, resistance, or impedance due to redox reactions occurring at the electrode or membrane surfaces, the electrochemical output can be immediately interpreted for diagnostic results^9,10^. All of these characteristics are important for the rapid mobilization of detection programs in response to an attack.

Although there have been many substantial reviews on the topic of bioweapons and bioweapon detection, such as the works published by Walper et al.^11^, Shah et al.^12^, and Rowland et al.^13^, there have been no substantial reviews on the use of electrochemical sensors. Without the presence of any review on the electrochemical detection of bioweapons, it was evident that we could fill a hole in the biosensing literature. We therefore present a review on the advances in electrochemical sensing of biothreats listed in category A by the Centers for Disease Control and Prevention (CDC). These agents are deemed to be the greatest risk for use as biological weapons due to their high rates of transmission and mortality (Table 2).

**Table 2:** Classification of potential bioterrorism agents (bacteria, virus, protozoan, and toxins) capable of inducing diseases in humans, according to the United States Centers for Disease Control and Prevention (CDC) Strategic Planning Group^5^

Category	Definition	Agent and disease
A	High-priority agents	*Bacillus anthracis* (anthrax)
	Easy to disseminate or transmit (person to person)	*Clostridium botulinum* (botulism, toxin)
	High-mortality rates	*Francisella tularensis* (tularemia)
	Potential for major public health impact	*Yersinia pestis* (plague)
	Cause public panic and social disruption	Variola major (smallpox)
	Special action for public health preparedness	Filoviruses (Ebola, Marburg)
		Arenaviruses (Lassa, Machupo)
		Bunyaviruses (Congo-Crimean, Rift Valley)
		Flaviviruses (Dengue)

## Results and discussion

### Electrochemical detection of bioweapons

#### Anthrax

*Bacillus anthracis*, a rod-shaped, gram-positive, spore-producing bacterium, is the causative agent of anthrax. Through the creation of potent exotoxins, *B. anthracis* targets vital systems and organs such as the cardiovascular system and liver, leading to rapid host death^14^. If a case is suspected in the United States, the Laboratory Response Network, an organization through the CDC, will be sent samples for rigorous testing, such as bacterial culturing, its susceptibility to specific bactericidal agents, and animal pathogenicity testing, all of which may take up to 72 h to complete^15^. Although 95% of anthrax cases are cutaneous, the inhalation of anthrax is largely concerning as a bioterrorism agent due to the difficulty in regulating airborne particulates; furthermore, death can occur in as little as 4 days after infection without treatment^16^. With test times taking 3 days and with the possibility of death occurring after 4 days, it is apparent that anthrax is an ideal candidate for the development of a rapid and precise method of testing.

#### Detection of *B. anthracis* via gold nanoparticles and electrodes

The use of gold nanoparticles (AuNPs) and gold electrodes in the sensing of biological materials is not a new concept and has been shown to display many advantages when used in tandem with electrochemical sensing. Together, there are three distinct advantages. First, an electrode employing AuNPs provides a much larger surface area, resulting in high current signals. Second, the biological chain can more intimately interact with AuNPs, thus facilitating the electron transport process. Finally, the sensitivity, selectivity, and robustness can be further enhanced^17^.

One recently developed method of anthrax detection uses the basis of DNA hybridization to detect a global gene regulator: the atxA gene, also known as the anthrax toxin activator gene. The complementary ssDNA to this gene is attached to gold nanoparticles, deposited on (3-mercaptopropyl)trimethoxysilane (MPTS), and then subsequently bound to a glassy carbon (GC) electrode for the creation of a DNA probe^18^. The ssDNA probe-modified electrode is incubated with the target gene for 40 min before being placed in methylene blue for 5 min, and DNA hybridization is subsequently monitored by differential pulse voltammetry (DPV). Although not rapid, the test is significantly quicker than those currently in use and has the distinct potential to be extremely user friendly.

**Fig. 1:**
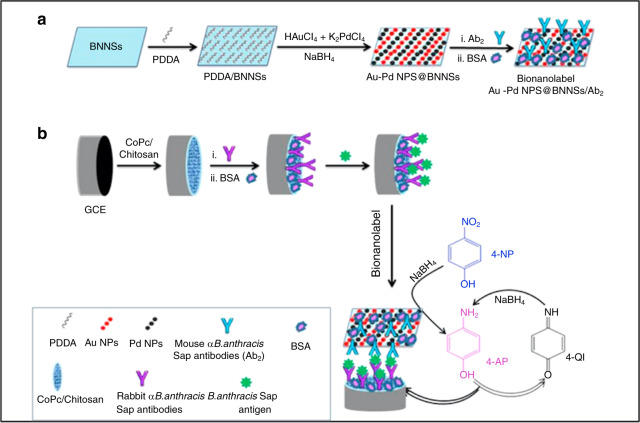
The creation of a nanoparticle based immunosensor for the detection of B. anthracis. Schematic representation of the synthesis of **a** Au–Pd NPs@BNNSs/Ab_2_ and **b** the proposed electrochemical immunosensor^20^

*B. anthracis* secretes two types of S-layer proteins, extractable antigen (EA1) and surface array protein (Sap). These novel biomarkers are released in growth medium during replication; no other known bacteria secrete this protein^19,20^. Boron nitride nanosheets (BNNSs), a structural analog of graphene, are considered a trailblazing material with many breakthroughs in the area of catalysis and biosensing^21,22^. The use of nanoparticles has also displayed an effectiveness in biosensing; it therefore makes sense that nanosheets and nanoparticles can be used in tandem for the creation of a novel electrochemical sensor for *B. anthracis*. Using *α-B. anthracis* Sap antibody-conjugated poly(diallyldimethylammonium chloride)-functionalized BNNSs (Au–Pd NPs@BNNSs/Ab_2_), 1 pg/ml Sap antigen is consistently and specifically targeted by antibodies and sensed using DPV^20^. On plate agar, *B. anthracis* can be identified within 1 h by the detection of Sap, thus providing a markedly quick way to detect *B. anthracis* during in-lab testing. This sensing platform is therefore highlighted as a cost-effective, sensitive, timely, and specific means to identify *B. anthracis* in environmental samples (Fig. 1).

Another advancement used for threat detection and prevention rather than diagnosis was proposed by Mazzaracchio et al.^23^. Their work described the creation of a miniaturized gold screen-printed, label-free aptasensor for *B. anthracis* spores using functionalized simulant *B. cereus* spores. The aptamer was immobilized on a gold screen-printed electrode; a response was then measured by the use of a portable instrument via electrochemical impedance spectroscopy (EIS). The test had a limit of detection (LOD) of 3 × 10^3^ CFU/ml and must incubate in the suspected sample solution for 3 h. The benefits of using aptamers rather than animal-based detection (such as antibodies) are that they are generally more stable, more cost-effective, and allow for early detection, as antibody species do not need to be developed. Although the proposed device does not provide immediate testing, the benefits of aptamer use, and the portability of the device make further exploration and development worthwhile.

In the work published by O’Sullivan et al.^24^, a single-pot reaction in combination with PCR amplification allowed researchers to simultaneously duplicate ferrocene-labeled dATP in the virulent DNA markers CAP and PAG. Once amplified, the product was deposited on an array of six modified circular gold electrodes and square wave voltammetry, with alternating potentials of 0–0.6 V, was applied. Limits of detection of 0.8 and 3.4 fM for CAP and PAG targets were found, respectively. The above work provides an extremely low LOD and can target multiple markers but does require amplification of the bacterial sample before detection can occur. This use of PCR restricts its use in testing; thus, this method is no more practical than the current standard in testing. However, the researchers are exploring the use of multiplex isothermal amplification and the use of a portable probe and potentiostat as a way to reduce costs and build upon the project.

#### Carbon-based electrodes and devices

Carbon nanostructures such as carbon nanotubes (CNTs), fullerenes, graphene nanosheets, and the additional use of carbon nanoparticles have been successfully used in a wide range of sensing platforms and continue to be used in new and novel electrochemical sensors^25–27^. Many organic sensors can be implemented on a platform for flexible and biodegradable devices and resorbable substrates. Carbon-based nanostructured materials, such as CNTs and graphene sheets, offer higher performance than other substances, such as silica, in terms of portability and sensitivity^28^.

Using a polypyrrole-functionalized carbon paste electrode, *B. cereus* spores (a simulant of *B. anthracis*) could be effectively imprinted and measured using cyclic voltammetry (CV)^29^. Through the use of a cetyl trimethylammonium bromide wash, the spores could be effectively removed from the sensor; thus, the sensor could be reused without compromising the integrity of the polymeric network, allowing for the repeated use of the sensor. Linear data collection was viable between 10^2^ and 10^5^ CFU/ml, showing its potential for use in real-world applications. The sensor was produced using simple polymerization techniques, further displaying a cost-effective means of production while remaining sensitive and rapid (requiring only 5 min of incubation time). Molecularly imprinted sensors are also extremely stable and can withstand environmental stressors, such as temperature, pressure, and pH changes. When stored at room temperature, they remain stable for several years postproduction. These sensors, when refined to be user friendly, should be at the front-line of monitoring *B. anthracis* attacks, particularly at postal service locations and other front-line agencies.

**Fig. 2:**
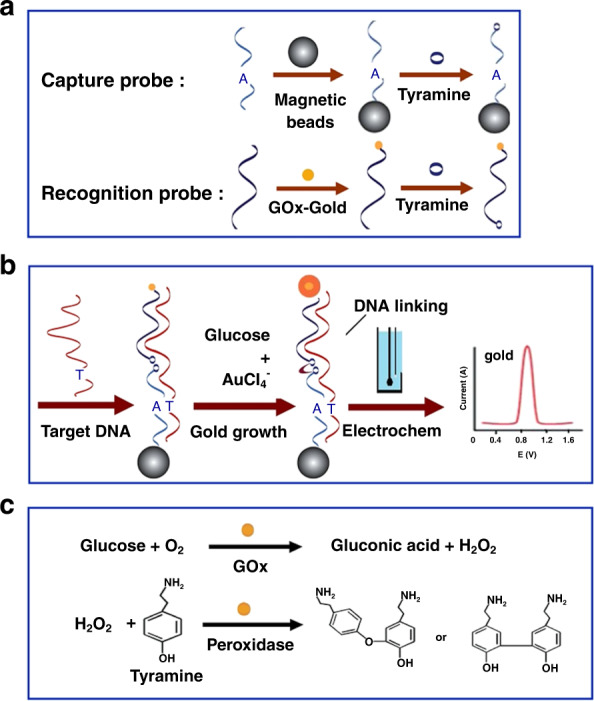
Schematic illustration of the principle and procedure for the electrocatalysis and mutation discrimination of DNA. **a** The loading of a DNA capture probe onto magnetic beads, the binding of a DNA capture or detection probe with tyramine, and the labeling of a DNA detection probe with GOx-Gold; **b** electrocatalysis steps, namely, DNA hybridization followed by the catalytic linking of DNA probes, gold growth, and electrochemical measurements on SPE; and **c** GOx-Gold catalyzed reactions for glucose oxidization and tyramine coupling^30^

Glucose oxidase (GOx)-doped gold nanoparticles were used to create a magnetic separation-based detector for anthrax in blood using a newly modified sandwiched electroanalysis method^30^. Using a tyramine linker, DNA fragments could bind and oxidize the present glucose to H_2_O_2_, thus facilitating reductive gold growth into anthrax enzymes. These signals could then be captured and graphed on a semi-log scale, exhibiting a detection limit of ~0.1 fM. This testing can be used to identify anthrax in human blood samples with ultrasensitive precision, but more must be done to understand the mechanism of this process. This process should also be tested against other common proteins and DNA fragments to determine its degree of selectivity (Fig. 2).

The creation of an electrochemical immunosensor for the sensitive, specific, and easy detection of anthrax protective antigen (PA) toxin in picogram concentrations was developed by Sharma et al.^31^ in 2015. Bismuth/heavy metal alloys were previously used for the creation of distinct stripping peaks, a broad cathodic potential range, and high sensitivity. The group then created a bismuth/Nafion-multiwall CNT composite. This composite worked in conjunction with titanium phosphate nanoparticle–cadmium ion-mouse anti-PA antibodies for heightened signal output from square wave voltammetry. The technique displayed a linear range from 0.1 to 100 ng/ml; furthermore, it boasted a detection limit of 50 pg/ml. If refined, the above work could be applied to allow for on-site testing, although challenges exist, such as the 35-min testing time needed for a conclusive result.

**Fig. 3:**
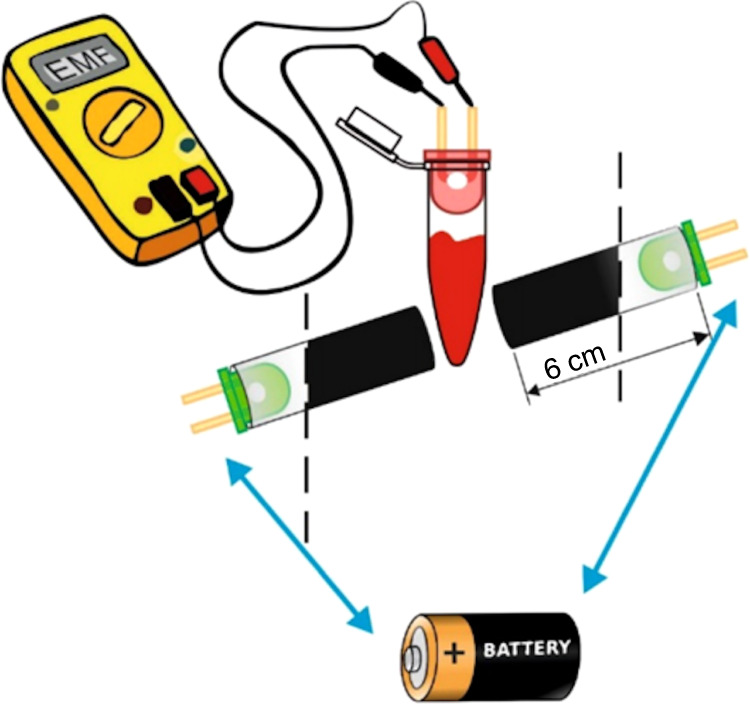
FDEDD device; two green LEDs placed coaxially in a 6 cm blacklight—impermeable tube and a red LED placed perpendicular to the axis. The sample is placed in the 0.2 ml PCR tube. The upper part of the PCR tube excellently suits the LED dimension, thereby ensuring no contact between the sample solution and LED. The internal photoelectric effect that generates diode electromotive force (EMF) is measured by a low-budget voltmeter (without any signal amplification)^32^

In the case of an attack, the rapid identification of the bioweapon is of the utmost importance. Ziółkowski et al.^32^ therefore proposed and developed a cost-effective and fully portable detection test for the *B. anthracis* pagA gene fragment, a fragment of the anthrax toxin translocating protein. The device uses graphene oxide (GO) nanoflakes to interact with pagA fragments to reveal a fluorescent marker once bound. Using a fluorometric-paired-emitter-detector-diode, this process takes only 2 min; furthermore, this process requires only mass-produced elements such as LEDs, PCR tubes, multimeters, and 2.5 V batteries. Although the detection limit of 0.625 μM is higher than that of spectrofluorophotometers, it is still sufficiently low when compared to PCR product detection methods. Due to the efficiency, cost, and portability of this test, the device could be made standard for environmental testing, along with its use in biosafety facilities and emergency testing (Fig. 3).

#### The future of detecting *B. anthracis*

We have found that the FDEDD device allows for the easy, timely, and portable detection of anthrax toxins, thereby promoting its use in security facilities. It is furthermore cost-effective and uses only commercially available materials. The development of other electrochemical fluorescent detection methods can continue to push the movement of such devices into the biosafety sector. The use of GOx-doped gold nanoparticles shows promise in allowing for point-of-care detection in human blood sera, an extremely important advancement for hospitals and individual diagnosis.

### Botulism

Today, botulinum neurotoxin, the product of *Clostridium botulinum* and the causative agent of botulism, is commonly used in cosmetic enhancement and medicine^33^. However, its destructive potency and heightened potential as a weapon still looms large. Although there have been no successful attempts to intentionally spread botulinum neurotoxin, it is still sanctioned as a Class A biothreat due to its potency and ease of procurement. With an estimated LD_50_ in humans of 1 ng/kg, botulinum neurotoxin is one of the most lethal substances known^34^.

The first case of research surrounding the use of botulinum neurotoxin occurred within Unit 731 of the Japanese biological warfare research unit during the Second World War^35^. Shortly thereafter, the USA was able to mass produce the toxin for use as a biological weapon. Since then, there have been three attempts to weaponize botulinum toxins by the Japanese cult Aum Shinrikyō^36^. In the early 1990s, the cult unsuccessfully disseminated large volumes of liquid botulinum toxin from trucks fitted with crude spray devices, deploying these in the vicinity of two U.S. Naval bases, the Narita airport, the Imperial Palace, and the headquarters of a rival religious group^37^. Fortunately, all these attacks failed; however, they illustrate how easily the toxin can be obtained and disseminated, hence the need for rapid and sensitive testing.

Currently, the standard for detecting the toxin is a mouse bioassay^38^. These assays involve the intraperitoneal injection of mice with a sample of the suspected toxin and subsequently observing each mouse for symptoms of the disease, such as a wasp-like waist and paralysis, over a 4-day period^39^. As one can imagine, this inelegant way of testing is both time consuming, uses live animal models, and would be inefficient in the case of a biological attack. We therefore outline some new adaptations and technologies that significantly reduce the time of testing from days to hours.

#### Identification of Botulinum neurotoxin via synaptosomal-associated protein, 25 kDa (SNAP-25) cleavage

Botulinum neurotoxins are zinc-dependent endopeptidases known to selectively cleave the soluble N-ethylmaleimide sensitive attachment protein receptor (SNARE) group of proteins^40^. In experiments conducted by Halliwell et al.^38^, two electrochemical methods were used: electrochemical impedance and a specialized combination of spectroscopy and CV. A gold electrode was then coated with a monolayer of the SNARE protein SNAP-25. This protein was cleaved by interacting with botulinum neurotoxins, and the resulting change in electrochemical properties was monitored using CV and EIS. The impedance biosensor was found to outperform the mouse bioassay in sensitivity, producing a response down to 25 pg/ml toxin and with a run time of <1 h. It is important to note that the samples must undergo processing before the test to filter out contaminating proteases; therefore, this method is not currently viable for point-of-care detection and would find much of its application in a security setting where detection is the primary concern. It should also be noted that pharmaceutical samples were the only tested group, so future tests should be performed on complex matrices, such as human serum.

Stemming off of the same group of researchers, Savage et al.^41^ took the detection of botulinum toxin a step further by the development of a SNAP-25 and vesicle-associated membrane protein (VAMP) sensor for the detection of botulinum neurotoxin serotypes A through E. Gold electrodes were used and SNAP-25/VAMP monolayers were yet again produced on the electrode surface before EIS was used to detect changes in the proteins. This time, concentrations in the fM range could be consistently detected. These samples were only laboratory species and were not conducted in complex matrices, nor were the samples separated from complex matrices. It would be recommended that the use of such matrices be tested in future studies.

**Fig. 4:**
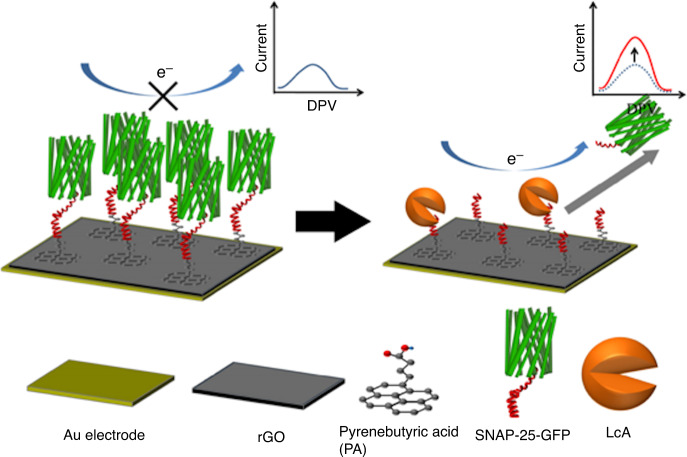
Schematic illustration showing the detection mechanism of rGO-based biosensors^42^

The use of SNAP-25 was again used by Chan et al.^42^ this time using a GO/gold electrode. SNAP-25 was grafted to the working electrode and detection was conducted via DPV. This sensor was demonstrated to have a linear working range from 1 pg/ml to 1 ng/ml and an LOD of 8.6 pg/ml. Perhaps the more interesting part of the experiment was the feasibility of detection in complex matrices, such as skim milk, thus potentially allowing for detection in liquids and foods (Fig. 4).

#### Botulinum neurotoxin detection via electrochemical immunosensing

**Fig. 5:**
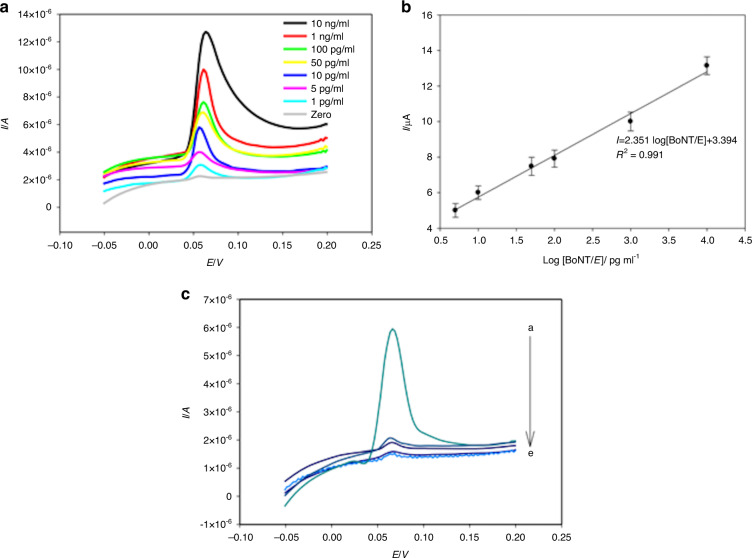
The efficiency of botulinum toxin detection using GNS/Ph–PhNH2/GCE detection. **a** Linear sweep stripping voltammetric peak currents against varying concentrations of BoNT/E in buffer solution; **b** concentration versus stripping current profile of GNS/Ph-PhNH2/GCE against botulinum toxins; **c** LSV responses on GNS/Ph–PhNH2/GCE against botulinum toxins E, A, B, F, and with no antigen from high to low, respectively^43^

The creation of an electrochemical immunosensor by Narayanan et al.^43^ involved the use of graphene nanosheet-aryldiazonium salt-modified glassy carbon electrodes (GCEs) as a sensing platform and the subsequent signal amplification using enzyme-induced silver nanoparticles (AgNPs) deposited on top of gold nanoparticles (AuNPs). The developed electrochemical immunosensor could detect botulinum toxin type E in a linear range from 10 pg/ml to 10 ng/ml with a minimum detection limit of 5.0 pg/ml and a total analysis time of 65 min. Although immunoassays are a less than ideal way of identifying toxins due to the cost of obtaining antibodies and their more complex user interfaces, the presented sensitivity is 5 pg/ml, which is the lowest to date for type E botulinum toxin. This sensor provides a method to detect botulinum toxin type E for prevention and security settings and could further provide useful information due to its low LOD (Fig. 5).

Botulinum toxin type A antibodies were immobilized on a GC electrode modified with Au nanoparticles/graphene-chitosan (AuNPs-Gr-Cs) for signal amplification purposes. Through the use of EIS, impedance was calculated, and changes were observed due to the resistance created at the redox probe. The impedimetric immunosensor developed by Afkhami et al.^44^ displayed a detection limit of 0.11 pg/ml and was furthermore able to detect toxins in human serum and milk, thus providing successful testing in complex matrices. Unfortunately, the electrodes could only be used over a 4-day period, as the signal rapidly decreased in efficiency after four consecutive days of use. If the lifetime of this electrode can be increased, it will be a welcome addition to the sensors used for botulinum toxin detection.

#### Proposed advancements for the detection of botulinum neurotoxin

Due to the integral involvement of SNARE proteins, it is unsurprising that SNAP-25 seems to be a promising protein to target for the detection of botulinum toxins. There is, however, a distinct lack of valuable testing using the SNARE proteins synaptobrevin-2 and syntaxin-1A. If we can implement all three proteins on an electrode, the impedimetric sensor should boast high levels of specificity and a low LOD, showing promise for use in environmental samples. The use of gold nanoparticles/graphene-chitosan also shows high potential for use in testing with complex matrices. Notably, creating covalent bonds between the electrode and detection molecules, such as using reactive carbon paper as the electrode, can increase the electrode life by significant proportions.

### Smallpox

Smallpox is caused by the major and minor Variola viruses and was the biological weapon of choice used by European colonists, the British during the American revolution, and the American confederates during the American revolution^5^. In Europe, during the eighteenth century, smallpox regularly killed between 200,000–600,000 people every year and reported fatality rates were between 20–40%^45^. As a bioweapon, the smallpox-causing Variola was used to target Indigenous Americans, causing a more than 50% decline in Indigenous American populations^46^. In more recent memory, as recent as the 1980s, the Soviet Union was able to mass produce liquid-form smallpox biological weapons^47^. Surprisingly, right around this time, smallpox vaccinations were terminated in most countries^48^. In fact, if used on contemporary populations, it was determined by Gani et al.^49^ that between three and five individuals of the present population would be infected by each active case post infection. With a history deep rooted in bioweaponry and a complete recession in vaccinations, smallpox remains one of the most feared and effective potential bioweapon agents in the modern age.

**Fig. 6:**
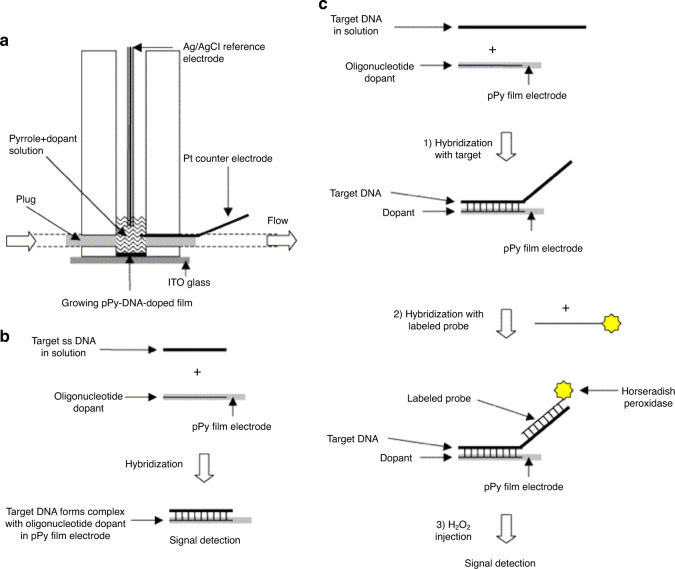
The proposed horseradish peroxidase-labeled probe and minicell for the detection of variola virus. **a** The electrochemical minicell and processes used for the preparation of DNA-doped conductive films (internal volume of 100μl, bottom opening of 3mm i.d.) and for target DNA chronoamperometric detection^50^; **b** direct detection of DNA hybridization and **c**, the indirect detection of DNA hybridization

Work conducted in 2005 by Komarova et al.^50^ used ultrathin DNA-doped polypyrrole films with oligonucleotides to achieve an LOD of 1.6 fmol per 0.1 ml, which is the lowest of any published research. Their work used chronoamperometry to achieve a one-step, reagent-free detection method that yielded results in seconds while being reusable and highly stable for storage. The use of an ultrathin film could be adapted to create a sensor that could be placed discreetly in areas where potential for biothreats is high, such as in ventilation systems. This work has not been built upon since (Fig. 6).

Research by Donaldson et al.^51^ explored the possibility of detection from throat-swabbed samples. Potential benefits of their work show that the virus could be detected in the oropharyngeal or respiratory mucosa before the infectious stage or the onset of illness, resulting in better prognosis and containment. Due to the low LOD of the polypyrrole probes proposed by Komarova et al.^50^, an adaptation for its use on throat swabs would streamline detection in cases of bioterrorism for individual cases.

### Plague

The most storied pandemic known to Europe is the plague, killing an estimated 30–50% of the European population^52^ and 33% of the estimated global population^53^. *Yersinia pestis*, the causative agent of plague, is widely considered one of the most likely pathogens to be used as a bioweapon^54^. In humans, the plague is a severe clinical infection that can progress rapidly despite antibiotic therapy and is associated with a high-mortality rate^55^. In fact, in its septicemic and pneumonic presentations, untreated cases are almost unanimously fatal^56^. Less severe but still presenting a significant mortality rate, untreated bubonic plague has a case fatality of 50–80%^52^. However, recovery rates are high if detected and treated within 24 h of symptom onset. We will therefore explore new simple techniques for the identification of *Yersinia pestis* through an electrochemical sensing platform.

**Fig. 7:**
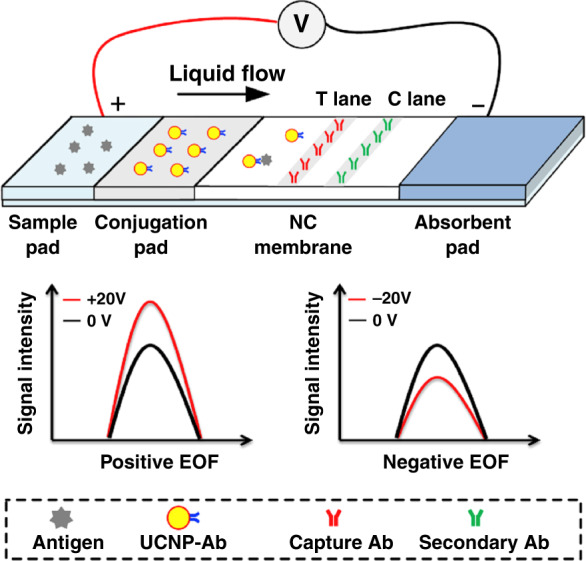
Schematic of the biosensor for the detection of plague. The electric field along the direction of fluid travel was defined as positive, as indicated by the arrow^57^

By harnessing the power of electrokinetic forces via the addition of electroosmotic flow, Zhao et al.^57^ were able to combine capillary-based immunochromatography assays with upconversion nanoparticles to increase the signal detection of *Y. pestis* by 64%. Although this work was not fully automated and required a scientific background to perform, it showed great future potential with a rapid assay time of 5 min and a detection limit of 1.2 × 10^4^ CFU/ml in soil samples. Thus, they demonstrated the potential for electrochemical techniques to enhance previously developed sensors (Fig. 7).

The use of EIS and label-free genosensing for detecting was performed by Komarova et al.^58^. The group created an assay using eight gold electrodes, some conjugated with specific binding sites for *Y. pestis* DNA detection, while others acted as nonspecific sites to act as a negative control. The assay could perform eight EIS measurements simultaneously in 15 min. The use of multiple electrodes was beneficial in confirming the reliability of detection. However, this study was not comprehensive and only illustrated the potential of the sensor. Thus, the LOD and specificity of *Y. pestis* should be further investigated.

#### Proposed advancements for the detection of *Yersinia pestis*

We feel that the work of Zhao et al. has the potential to be translated into diagnostic and point-of-care applications. Experimentation using similar methods in blood serum would be beneficial to this effect.

### Viral hemorrhagic fevers

Viral hemorrhagic fevers (VHFs) are a group of illnesses caused by four different families of viruses: arenaviruses, filoviruses, bunyaviruses, and flaviviruses^59^. Due to the epidemic causing the potential of VHFs, there has been an increased interest in VHF pathogens^60^. As global temperatures continue to increase, the potential for VHFs to thrive on a global scale also increases^61^. The heightened capability for human-to-human transmission and high levels of mortality call for quick, fast, and reliable identification. We will therefore explore the means of the simple electrochemical identification of these easily transmittable and high-mortality diseases (Table 3).

**Table 3:** Types of VHF, modes of transmission, virulence, therapy, and mortality^62^

Virus	Genus	Transmission	Virulence	Isolation	Treatment	Mortality %
CCHF	Bunyaviridae	Tick	Moderate	Yes	Ribavirin	10–50
Dengue	Flaviviridae	Mosquito	Low	No	Supportive	1–20
Ebola	Filoviridae	Primate	High	Yes	Supportive	50–90
Marburg	Filoviridae	Primate	High	Yes	Supportive	50–90
Hanta Virus	Bunyaviridae	Rodent	Low to moderate	Extremely rare	Ribavirin	5–15
Lassa	Arenaviridae	Rodent	Moderate	Uncommon	Ribavirin	30
RVF	Bunyaviridae	Mosquito	Low to moderate	No	Ribavirin	1% RVHF, 50% die
SAHF	Arenaviridae	Rodent	Moderate	No	Supportive	30
Yellow fever	Flaviviridae	Mosquito	Moderate	No	Supportive	25–50

#### Ebola

Ebolaviruses are negative strand RNA viruses that belong to the Filoviridae family. These public health pathogens are primarily transmitted by human-to-human contact with infected body fluids and corpses and cause severe and acute systemic disease with extremely high-mortality^63^. Ebolaviruses have substantial epidemic potential, as shown by the 2013–2016 West African outbreak^64^. This outbreak was unprecedented in scale, with more than 28,000 confirmed cases and 11,000 deaths^65^. Although there is no previous use of Ebolavirus as a biological weapon, its ease of transmission, high rate of mortality, and difficulty of containment make it a potent biological agent if weaponized. The rapid and simple electrochemical detection of Ebolavirus is therefore essential in the case of a weaponized attack.

**Fig. 8:**
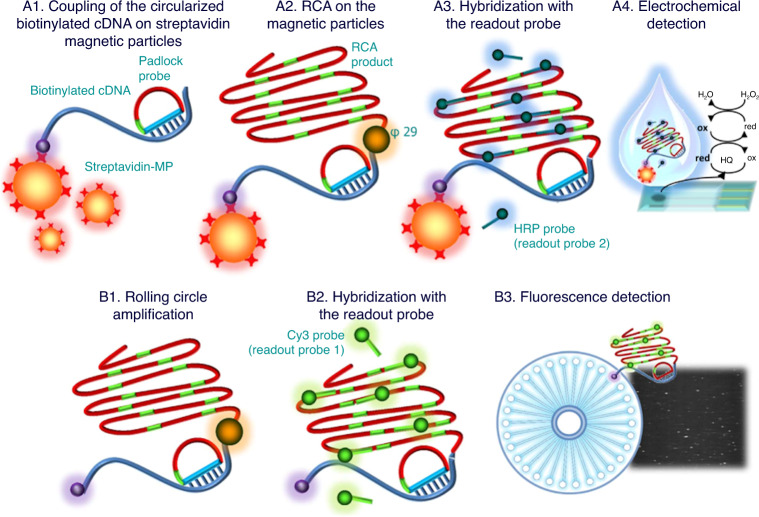
Schematic representation of the electrochemical genosensing of Ebolavirus cDNA by rolling circle amplification. **A1** Coupling of the circularized biotinylated cDNA (from 200 zmol up to 20 amol) on streptavidin magnetic particles for 5 min at RT. **A2** RCA on the streptavidin-MPs using φ29 DNA polymerase for 60 min at 37 °C. **A3** Hybridization with readout probe 2 labeled with HRP for 15 min at 37 °C. **A4** Enzymatic reaction (with H2O2 as a substrate and hydroquinone (HQ) as a mediator) and electrochemical determination on screen-printed electrodes by square wave voltammetry at a potential ranging from 0 to −0.7 V. Schematic representation of the Aquila-amplified single-molecule counting procedure. **B1** RCA using φ29 DNA polymerase for 60 min at 37 °C. **B2** Hybridization with readout probe 1 labeled with Cy3. **B3** Counting of Cy3-labeled RCA products with an Aquila-amplified single-molecule counter (Q-linea)^67^

There is a promising sensor for use in underdeveloped and rural areas that meets the criteria for affordable, sensitive, specific, user friendly, rapid and robust, equipment free, and deliverable to end users (ASSURED) testing recommended by the WHO^66^. This test detects the *L gene* sequence that is present in the five most common Ebola strains. Padlock probing is used to bind target DNA with magnetic particles that then undergo isothermal amplification and are monitored using square wave voltammetry on commercially available screen-printed electrodes. The sensor has an LOD of 33 cDNA molecules, making it extremely sensitive^67^. The assay takes a total time of <2.5 h, making it relatively rapid. Isothermal amplification can also be modified to occur at room temperature, making this an ideal test in areas with minimal lab access. To prepare this test for use in industry, the work should be repeated using complex human matrices rather than a cDNA mimic of the virus (Fig. 8).

Ilkhani et al.^68^ again used cDNA to detect Ebolavirus. The surface of a gold screen-printed electrode was modified with complementary ssDNA. When in contact with Ebola, hybridization occurred, and the hybrid was conjugated with a streptavidin-alkaline phosphatase for use in redox processes to facilitate electrochemical measurements. The process was monitored through both EIS and DPV testing. The total detection time was optimized at 60 min. This sensor is not only more rapid than the previous sensor but also requires no amplification of DNA.

Another method of detection for the five most common species of Ebola involves using a field-effect transistor with reduced GO, as the conducting channel, and anti-Ebola probes immobilized on the surface^69^. This sensor detects the surface glycoprotein that is found in all five common Ebolaviruses. The results are both instantaneous, sensitive, and specific with an LOD of 1 ng/ml. The sensor was tested in a complex matrix containing PBS buffer, human serum, plasma, and Ebola glycoprotein to mimic patient samples. Thus, this sensor is a candidate for rapid and early testing; however, more work needs to be done to improve the sensor function, particularly in patient samples.

In a paper by Yuhai et al.^70^, a rapid and sensitive detection method for Ebolavirus is made using electroluminescent nanospheres (ENs) to provide an ~85-fold improvement in electroluminescent signaling. The group also added magnetic nanobeads (MBs) to selectively separate Ebola targets in complex media, thus providing another threefold increase in signaling. ENs were constructed by embedding CdSe/ZnS quantum dots into poly(styrene/acrylamide) copolymer nanospheres. These ENs were then attached to polyclonal antibodies and put into solution with Ebolavirus samples. CV was run from −1.65 to 0 V, and a 900 V photomultiplier tube was used for ECL recording. A LOD as low as 5.2 pg/ml Ebolavirus could be detected in 2 h.

The final sensor is the only one to have used samples from patients^71^. It is rapid, affordable, and specific. This method also uses an Ebola padlock probe for the *L gene*. cDNA derived from the clinical RNA samples was synthesized using rolling circle amplification onto magnetic beads. These cDNA samples were then labeled with GOx. The sample was then placed on a screen-printed carbon electrode with Prussian blue, followed by the addition of glucose. A chemical reaction occurred, and H_2_O_2_ was produced and subsequently reduced. The reduction was measured using chronoamperometry. Although preparation of this sensor and sample is time consuming, the analytical time is <10 min, making it a great point-of-care alternative.

#### Marburg

Among VHFs, Ebolavirus, and Marburg virus, which are hemorrhagic fevers of the family filoviridae, are considered the most lethal; these can have a fatality rate of up to 90%^72^. The CDC recommends the following minimum biosafety and biocontainment requirements that should also be applicable in low-resource settings: the patient should be isolated, preferably in a single room with an adjoining toilet and separate anteroom as a changing area for hospital personnel; personnel must wear a scrub suit, gown, and apron, two pairs of gloves, a mask, headcover, eyewear, and rubber boots; all non-disposable material that has come into contact with the patient should be cleaned with soap and disinfected with bleach; waste should be disposed of in a place set aside for this purpose and burnt at least daily; laboratory workers working with patient material should wear protective clothing; and bodies of deceased patients should be sprayed with bleach, sealed in a body bag that is also sprayed with bleach and buried with the same precautions as are taken in the hospital^73^. Unfortunately, although Marburg viruses are highly fatal and severely contagious^74^, there exists a distinct lack of electrochemical detection of Marburg virus (Table 4).

**Table 4:** Clinical and laboratory features of Marburg hemorrhagic fever^72^

Clinical features
Fever, chills, relative bradycardia
Headache
General malaise, anorexia
Sore throat, dry cough, chest, back, joint and muscle pain
Nausea, vomiting and diarrhea
Lymphadenopathy
Right upper abdominal pain or tenderness
Conjunctival injection
Maculopapular or popular rash (from day 5)
Mucosal bleeding, oozing from puncture sites
Septic shock
Lethargy, drowsiness and dysaesthesias
Hepatic encephalopathy
Orchitis (late complication)
Uveitis (late complication)

#### Yellow fever

A flavivirus transmitted by mosquitos, yellow fever (YF), is noncontagious, but has no specific treatment^75^ and a fatality rate ranging from 25 to 50%^62^; thus, its potential as a bioweapon is still high. Vaccination to the disease is only recommended when staying in areas where it is endemic^76^, meaning that the majority of Western society has no immunity to the disease. Almost all cases of YF in North America are imports; however, with increasing temperatures due to climate change, the habitat of the vector is increasing^77^. The intentional release of YF vectors could therefore make a permanent home in parts of North America and Europe and become endemic, as seen in Brazil^78^.

At the time of writing, no electrochemical sensors have been developed for the detection of YF, but there is potential, as work in other flaviviruses is ongoing^79–81^. Whereas current methods of detection struggle with cross-reactivity in confirmative diagnosis^75^, an electrochemical sensor would be of great benefit.

#### Other VHFs

The electrochemical sensing of other VHFs is critically under-researched despite the increasing threat of disease vectors becoming permanent fixtures in a warming climate. If weaponized, the distinct lack of sensing poses increased challenges in the rapid mobilization of large-scale testing.

#### A call for research focus in the detection of VHFs

Due to the ever-increasing global temperatures and following the increased potential for VHF transmission, it is prudent that ready detection and diagnosis of these deadly viruses become standard. Viruses have already shown potential for mass destruction and death. We therefore call for an increase in VHF detection platforms for quick, selective, and effective identification.

### Tularemia

Discovered in 1911 by George McCoy while investigating a supposed bubonic plague in squirrels, *Francisella tularensis* and the disease tularemia was shown to be a highly contagious and infectious illness^82^. The disease also displays various mechanisms and targets multiple tissues. The most likely to be weaponized is oropharyngeal and gastrointestinal tularemia, which can develop after oral exposure and displays a fatality rate of up to 60%^83^. These characteristics led to studies of its potential as a biological weapon, including involuntary human experimentation by Japan among civilian, political, and military prisoners. The end goal was for use in warfare during the Second World War^84^. Later, in the United States, voluntary human experimentation occurred in the 1950s–1960s with penitentiary inmates and noncombatant soldiers^82^. Soviet Union scientists allegedly developed a vaccine-resistant strain, which they tested as a biological weapon in 1982–1983^85^. Because it has a high aerosol-related infection rate, low infectious dose, and the ability to induce fatal disease, *F. tularensis* is considered a potential agent of biological warfare.

The most recent electrochemical advancement in detecting *F. tularensis* was the fabrication of an electrochemiluminescence (ECL) immunosensor by grafting monoclonal antibodies onto screen-printed gold electrodes with automated monitoring^86^. The sensor used the active luminophore Ru(bpy)_3_^2+^ to detect fragments of the envelope at an LOD of 0.4 ng/ml, while an LOD of 70 bacteria/ml was observed when using whole antibody capture, and 45 bacteria/ml when the whole bacterial cell was targeted. The group also tested for specificity against *Yersinia enterocolitica*, although it would be further advised to test against other common bacteria.

**Fig. 9:**
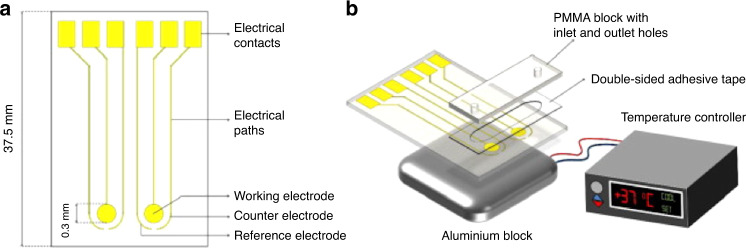
Design of the sensing platfom for the detection of tularemia. **a** The electrode with its respective counter and reference electrodes and **b** ensemble with its electrodes, PMMA block, aluminum block, and temperature controller^87^

Del Río et al.^87^ also featured gold electrodes in their work on the detection of *F. tularensis*. By using the forward primer Ft FwP for *F. tularensis*, the primer was immobilized on a gold sensor via the use of a thiol or amino moiety, elongated using recombinase polymerase amplification and then bound with a horseradish peroxidase label. Chronoamperometry was used to detect oxidized tetramethylbenzidine, an indication that the horseradish peroxidase label had bound to the target molecule. The assay could be performed in approximately an hour and had an LOD of 10^4^ DNA copies/µl. However, the overnight incubation period, lack of mobility, and mandatory use of laboratory space currently hinder this work for quick and easy testing (Fig. 9).

**Fig. 10:**
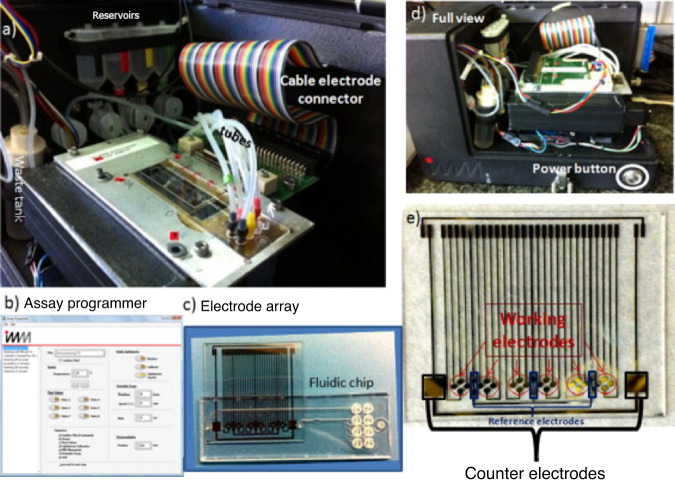
Amperometric immunosensor detection setup. The setup contains a peristaltic pump positioned behind the reservoirs to provide solution flow into the electrode array mounted within the microfluidics. **a** Electrode array with microfluidics placed in the platform and connected to the potentiostat for amperometric measurement; **b** a sample script-based assay program; **c** electrode array integrated with microfluidics; **d** a full front view of the prepared testing device; and **e** lithographically produced gold electrode array with internal reference electrodes and counter electrodes^88^

Automated sensing is optimal for use in a biothreat situation, as its simplicity results in ease of training in regard to large-scale testing. Work done by Dulay et al.^88^ resulted in the creation of a fully automated sensing platform using gold electrodes and microfluidics to assist in automation. The only hands-on time is the addition of sample, with a complete run time of <20 min. The sensor detects the lipopolysaccharide found on the bacterial membrane of *F. tularensis*. This was first done by grafting anti-*Francisella* antibody FB_11_ fragments onto the gold-plated electrode and subsequently measuring loose lipopolysaccharide and whole cell *F. tularensis* using amperometric techniques. Whole antibodies were then grafted onto the electrodes and tested using the same methods. The antibody fragments retained 85% antigen recognition under proper storage over 45 days. A LOD of 38 bacteria/ml was found. The writers report no cross-reactivity and hope to further this platform to perform multiplex detection in real-world applications, such as its implementation in ventilation systems (Fig. 10).

#### Proposed advancements for the detection of Tularemia

The use of automated sensing platforms is a welcome addition to any facility where testing is required. Not only does automation free the hands of researchers, but a testing time of ~15 min is very convenient. We encourage researchers to miniaturize the testing apparatus and use anti-*Francisella* antibodies and antibody fragments on a chip-like electrochemical sensor. These electrochemical sensors will allow for more affordable and accessible testing platforms.

### Combined sensing

The optimal form of sensing for the detection of biothreat agents is a combined sensing platform for the environmental testing of multiple biological weapons at once. These combined platforms would allow for the specific identification and differentiation of a bioweapon via the use of a single test in a timely manner. Thus, these combined platforms would allow for a quick and safe response for security, community, and government locations, along with healthcare systems. Combined systems would also further reduce the cost of buying multiple sensing platforms, reduce the requirement for multiple heavy, unportable machinery, and allow for simplified training.

**Fig. 11:**
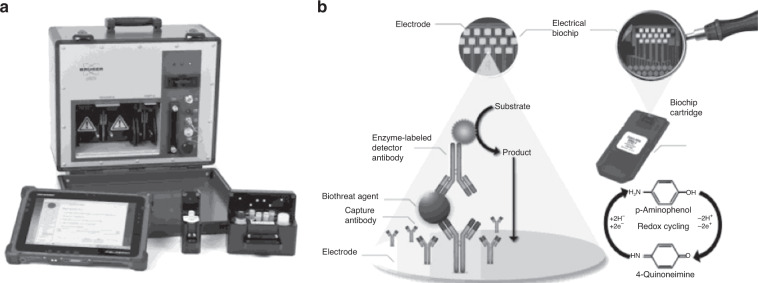
The pBDi system. **a** pBDi system including reagents and sample holder with ruggedized tablet PC. **b** Principle of biothreat agent detection using electrochemical ELISA technology^89^

Most recently, portable BioDetector integrated (pBDi), a portable biothreat detection unit approximately the size of a small suitcase, has been developed to detect four Class A biothreats: *Y. pestis, B. anthracis, F. tularensis*, and *orthopox* viruses (making it one of the few electrochemical sensors for smallpox)^89^. Using gold electrodes conjugated with targeted antibodies, the system enables the specific binding of an antibody to its subsequent antigen. These agents are then quantified by measuring the electrical current of the respective enzymatic redox reactions on the electrode surfaces. The reaction is further amplified via redox cycling, thus increasing the sensitivity of the electrodes. The detection platform is compact and has been developed for ease of use with nonscientific personnel and minimal sample preparation. In addition to a processing time of ~20 min and an LOD of 10^4^ plaque forming units per milliliter, this method shows potential for point-of-care and environmental detection; however, further testing should be performed on complex matrices containing human serum so that point-of-care testing can take place (Fig. 11).

Another combined electrochemical sensor detects *B. anthracis, F. tularensis*, and smallpox based on PCR and amperometric detection^90^. With an assay time of 8 min, this work shows considerable promise; however, in its current state without full automation and a narrower group of detectable diseases, we believe it to be inferior to the previously mentioned pBDi system.

Another combined sensor using ECL and DNA sensing has been developed but is limited to only *B. anthracis* and *F. tularensis*^91^. In addition, the system is not automated, making it difficult for use in large-scale testing.

### Future of the electrochemical sensing of bioweapons

We propose that the ideal platform should be relatively inexpensive, portable, selective to many bioterrorism agents without the creation of false positives or negatives and should boast a low LOD for each bioagent. The WHO’s recommendation for ASSURED testing is critical to the efficient advancement of bioweapon detection. With these changes, we hope that there will be further improvement in the detection, ease of use, reusability, and portability of electrochemical sensors.

The sensitivity and LOD of the most promising electrochemical detection methods targeting bioweapons are listed in Table 5. In summary, the best performing electrochemical sensor targeting *Bacillus anthracis* has been developed using a GOx-modified gold nanoparticle electrode^30^ that demonstrates an LOD comparable to or even lower than that of the conventional HCR technique and fluorescence spectroscopy, respectively. The proposed sensing platform has distinctive advantages over other electrochemical sensor prototypes in terms of its amplification of electrical signals and selectivity. The proposed sandwich detection approach enables the ultrasensitive electroanalysis of anthrax DNA present in blood as well as discrimination against targeted DNA with a single-base mutation. The recommended approach for electrochemical detection of Botulinum neurotoxins would be the development of an impedimetric sensor opposed to voltammetry or amperometry signaling. Not only is impedance more sensitive, but it allows for the identification and separation of Faradaic and non-Faradaic processes occurring at the electrode. The proposed bioelectrode^41^ can detect active toxins at concentrations as low as 25 fg/ml, which is lower than the standard mouse bioassay. However, this biosensor was only tested in buffer solution; therefore, more work is needed to further verify its selectivity in real-world samples, such as within bodily samples or food. The electrochemical detection of viral smallpox DNA showed very good sensitivity at the level of 1.6 fmol/0.1 ml. What should be highlighted as a benefit of this electrochemical sensor is its short analysis time, as it demonstrates a direct response to target DNA. Compared to indirect assays that necessitate electrochemical labels and reagent-intensive amplification, the proposed electrochemical method is reagent-free and detects DNA in one-step. The response time of this bioelectrode is counted in seconds, which is much faster than the conventional method of detection of the same target DNA with similar sensitivity. The electrochemical sensing of *Yersinia pestis*—the causative agent of plague—has a significant advantage over conventional ELISA, as it is much faster and displays comparable or even better sensitivity. The proposed electrochemical platform^57^ uses an electroosmotic flow in conjunction with an immunochromatographic assay, resulting in a rapid response. The recent development of an electrochemical detector of Ebolavirus is of great importance because it demonstrates good sensitivity and a short response time without time-consuming sample pretreatment or use of sophisticated instrumentation^70^. The proposed sensing platform is unique and combines CdSe/ZnS quantum dots acting as an electroluminescent signal amplifier with MBs that selectively separate targets from complex samples. This method simplifies the operation process and saves time. The proposed electroluminescent platform showed good reproducibility and specificity and is highly promising for further development toward clinical diagnostics.

**Table 5:** Selected sensor parameters for electrochemical detection methods (sensitivity, limit of detection) of bioterrorism agents categorized as class A according to the CDC

Target agent	Sensing platform	Linear range	Detection limit (LOD)	Refs.
Anthrax				
Electrochemical detection	DNA-modified gold NP/GC electrode	10^−11^–10^−9^ M	1 pM	^18^
	Antibody-conjugated boron nitride	5–100 mg/ml	1 pg/ml	^20^
	Array of gold electrodes	10 aM–100 pM	0.8 fM	^24^
	Polypyrrole-carbon paste electrode	10^2^–10^5^ CFU/ml	10^2^ CFU/ml	^29^
	Glucose oxidase-modified gold NP/GC electrode	0.2–6.4 fM	~0.1 fM	^30^
	BiNPs/Nafion-MWCNTs/GCE	0.1–100 nM	50 pg/ml	^31^
	Graphene oxide-based electrode	0.625–2.5 μM	0.625 μM	^32^
Conventional examples	Fluorescent analysis	2 pM–0.02 μM	6.63 pM	^92^
	Hybridization chain reaction (HCR)	0.20 pM–0.50 μM	1 fM	^93^
Botulism				
Electrochemical detection	Gold electrode-SNAP-25 protein	375–750 pg/ml	25 pg/ml	^38^
	Gold electrode-SNAP-25 protein	25–125 fg/ml	25 fg/ml	^41^
	GO-gold electrode- SNAP-25 protein	1 pg/ml–1 ng/ml	8.6 pg/ml	^42^
	Graphene-aryldiazonium salt-glassy carbon electrode	10 pg/ml–10 ng/ml	5 pg/ml	^43^
	Gold–graphene–chitosan	0.27–268 pg/ml	0.11 pg/ml	^44^
Conventional examples	ELISA	<100 pg/ml	2 pg/ml	^94^
	Mouse bioassay	>0.02 pg/ml	10 pg/ml	^95^
Smallpox				
Electrochemical detection	DNA-modified polypyrrole	NA	16 fmol/ml	^50^
Conventional examples	PCR	1 pg/ml–1 μg/ml	NA	^96^
Plague				
Electrochemical detection	Antibody-modified copper electrode	NA	1.2 × 10^4^ CFU/ml	^57^
Conventional examples	ELISA	NA	3 × 10^4^ CFU/ml	^57^
VHF—Ebola				
Electrochemical detection	FET-modified with antibody	1–444 ng/ml	1 ng/ml	^69^
	CdSe/ZnS quantum dots/copolymer nanospheres	0.02–30 ng/ml	5.2 pg/ml	^70^
Conventional examples	Chromatography	NA	150 ng/ml	^97^
VHF—Tularemia				
Electrochemical detection	Screen-printed gold electrodes	<20 ng/ml	0.4 ng/ml	^86^
Conventional examples	ELISA	<27 ng/ml	27 ng/ml	^98^

For comparison, sensor parameters for conventional analytical methods are reported for the same agents

The works by Pöhlmann et al.^89^ established a great foundation for the future of biosensing platforms. These platforms are extremely portable and detect four different Class A bioweapons. If researchers are further able to miniaturize these sensing platforms, allowing them to be transported as kits, the rapid establishment of medical testing sites and medical personnel will be increasingly feasible. It would be of great benefit to tailor these sensing kits for the ability to test complex matrices, such as blood, saliva, and mucous, and environmental samples, such as soil and water.

We further stress that the development of simple electrochemical VHF sensors is paramount to a safe and prepared tomorrow. Especially as global temperatures increase, the ability of VHF vectors, such as ticks, mosquitoes, and others to deliver disease only increases^61,99,100^. If not used for detecting bioweapon agents, these sensors could be used in the detection of disease. Screen-printed and paper electrodes also display distinct advantages in terms of portability, cost, and ease of use when conducting electrochemical sensing. If possible, these screen-printed or paper electrodes could be beneficial for use in underfunded communities with limited resources.

As time progresses and as history indicates, it becomes increasingly likely that bioweapons will be used not only again but also to a larger degree of success than before. It is apparent that the use of bioweapons in war or in bioterrorism could cause much stress to an established community. We were able to discuss some of the most prolific Class A bioweapon agents and their detection in the environment and clinical studies. If the environment is tested after an attack, the treatment for patients can be accelerated, and proper infrastructure can be erected. For more isolated incidents, such as the mailing of bioweapons, testing units should be available and utilized in biosafety and shipping facilities worldwide. In either case, a rapid and standardized testing regimen for bioterror agents could save not only individuals but also entire cities.

## Supplementary information

combined copyright permissions for all figures and tables
